# Department of Practice of Medicine

**Published:** 1895-08

**Authors:** 

**Affiliations:** Galveston


### Current Medical Literature.

### DEPARTMENT OF PRACTICE OF MEDICINE.

EDITED BY PROF. ALLEN J. SMITH, M. D., GALVESTON.

DIAGNOSIS IN ŒSOPHAGEAL DISEASE.—Dr. Bayard Holmes, of Chicago (*Medical News*, May 25, 1895), suggests, in order to obtain an idea of the nature of œsophageal obstructions, that an œsophageal bougie be coated over at the tip, or below the olive along the handle, with a small mass of dental wax which is soft enough to take impression of such obstruction after having been warmed to 120° F., and which at 98° F. will be hard enough to retain the impression. Several cases, illustrative of its value in recognizing the nature and exact position, are mentioned in the paper.

TREATMENT OF NEUROSES IN EARLY SYPHILIS (*Medical Standard*, April, 1895).—Dr. Frank Lydston, of Chicago, calls attention to the need of nervous prophylaxis at all stages of syphilis, but especially during the early stages. After recommending a discontinuance of alcoholics and tobacco, because of their toxic effects on nerve protoplasm, and hence as being strong predisposing causes to brain and nervous diseases in syphilis, he writes very urgently in favor of the use of certain of the gold preparations. He found the chloride of gold and sodium of special value, as a tonic and alterative, in cases with a tendency to nerve involvement. Later, he has preferred to use the bromide of gold and arsenic, or the bromide of gold and mercury, instead of the sodium salt, and with gratifying results. Gold seems to have a special value in preventing sclerotic changes in the tissue affected by the syphilitic formation. The bromide in the combination may be of service in correcting vaso-motor perturbation.

MALARIAL PSEUDO-TUBERCULOSIS.—Duba, writing originally in the *British Medical Journal*, is quoted in the *Medical and*

*Surgical Reporter*, February 16, 1895, as describing such a condition as not infrequent in malarial countries, occurring in persons who have had malaria for a long time. It manifests itself by an oncoming weakness, anorexia, emaciation, a gradually-developing dry, hacking cough, some dyspnœa and irregularities in the temperature, especially toward night. Hæmoptysis sometimes occurs. Some evidence of consolidation may be made out at times, on physical examination of the lungs; but examination of the sputum fails to discover tubercular bacilli. The cases recover under quinine and arsenic. It is thought possible that from vascular obstruction from accumulation of the pigments of the blood about the slow areas of circulation of the apices, local pneumonic processes, with consolidation, occur.

---

APPENDICITIS (*Medical and Surgical Reporter*, February 16, 1895).—Dr. J. C. Lange, of Pittsburg, in a paper upon this subject, states as his belief that the old idea of the causation of inflammation of the appendix, with the development of gangrene, or suppurative change, is erroneous in attributing it usually to the presence of foreign bodies or fæcal accumulations in the lumen of the blind gut. He regards the constant motion to which the appendix is subjected by the peristaltic movements of the intestine, and its own peristalsis, together with the low state of appendiceal nutrition (due to its relatively poor blood supply), as the main predisposing influences. These together produce a low resistive power of its tissues, permitting their penetration by the ever present *bacillus coli communis*. This micro-organism is capable of inducing exudative and formative inflammations, but without the assistance of the pyogenic micrococci can not induce suppurative disturbance, and hence could, if alone, cause nothing more serious than thickening of the appendiceal walls, obliteration of the appendix, or appendiceal adhesions. The author concedes, at the outstart of his consideration of the treatment of appendicitis, that every abscess of the appendix requires surgical operation, and that in all cases of doubt operation is indicated. But he believes the medical treatment, directed to prevent the formation of abscess, as of the utmost importance; and he does not agree with those who would decry any but surgical interference, even in early appendicitis, upon the ground that even if the inflammation be ameliorated and apparently prevented, the continued existence of the appendix in the human body is sufficient menace of recurrence to warrant its extirpation. Dr.

Lange would direct his treatment entirely toward preventing the action of the *bacillus coli communis* and of the suppurative micrococci, and, as far as possible, the accession of the latter to the focus of inflammation. This last measure is sought to be attained by quieting, as much as possible, for a period, the peristaltic movements of the bowel, putting the bowel in splints, as it were, by means of morphia; and to gain the former end the author would administer, as a germicide, in doses less than purgative, the mild chloride of mercury. With the latter, as local intestinal antiseptics, guiacol, iodoform, the subgallate of bismuth, and charcoal, have been used, apparently with benefit, in some of the cases reported by the author. He regards the older methods of purgation as distinctly contraindicated, as causing motion and further irritation of the inflamed appendix. Dr. Lange bases this method of medicinal treatment of rest and destruction of the germs, upon a series of eighteen cases, in all of which recovery without abscess formation occurred.

---

INSUFFLATION OF SODIUM CHLORIDE INTO THE NASAL CAVITY FOR RELIEF OF PAIN.—Dr. W. M. Capp (*Medical News*, June 15, 1895) recommends highly the insufflation, through an ordinary insufflator, or other appropriate tube, of from two to four grains of pulverized table salt, as a measure tending to give immediate relief in facial pain or headaches arising from trifacial irritation from decayed teeth, eye-strain, or from other causes, such as ear affections, hysteria, or uterine reflexes. The measure was first applied, according to the author, by Leslie, and published in the *Edinburgh Medical Journal*, January, 1890; the latter had successfully employed it in the treatment of obstinate and long standing, as well as acute neuralgia, headache, faceache, earache, toothache, and bronchial asthma. The application causes about the same temporary discomfort as would a pinch of snuff, but is not followed by bad results, and is usually successful.

